# Uveal and retinal abnormalities in an Asian neurofibromatosis type 1 cohort: a cross-sectional study with age-stratified analysis

**DOI:** 10.1186/s40662-026-00488-y

**Published:** 2026-05-13

**Authors:** Shipeng Guo, Xuan Yu, Haonan Ma, Mingyang Wang, Dian Jiao, Xuerui Zhang, Yuan Yang, Haodong Xiao, Wenting Zhang, Huanyu Liu, Yufan Yang, Jie Peng, Zhichao Wang, Peiquan Zhao

**Affiliations:** 1 Department of Ophthalmology, Xinhua Hospital Affiliated to Shanghai Jiao Tong University School of Medicine, No.1665, Kongjiang Road, Shanghai, 200092 China; 2 Department of Plastic and Reconstructive Surgery, Shanghai Ninth People’s Hospital, Shanghai Jiao Tong University School of Medicine, No.639, Zhizaoju Road, Shanghai, 200011 China; 3 Neurofibromatosis Type 1 Center and Laboratory for Neurofibromatosis Type 1 Research, Shanghai Ninth People’s Hospital, Shanghai Jiao Tong University School of Medicine, Shanghai, 200011 China

**Keywords:** Neurofibromatosis type 1, Ocular manifestations, Lisch nodules, Choroidal abnormalities, Asian cohort

## Abstract

**Background:**

Although ocular manifestations of neurofibromatosis type 1 (NF1) have been described extensively in Western cohorts, systematic data in Asian populations remain limited, and age-related phenotypic variations have not been comprehensively characterized. This study investigated the prevalence and multidimensional characteristics of NF1-related ocular manifestations in Asian populations, using age-stratified analysis of uveal and retinal abnormalities.

**Methods:**

In this cross-sectional study, 228 Chinese patients with NF1 underwent comprehensive ophthalmic evaluations. Examinations included slit-lamp biomicroscopy, ultra-widefield (UWF) fundus photography, near-infrared reflectance (NIR) imaging, and optical coherence tomography (OCT) to assess retinal and uveal abnormalities.

**Results:**

A total of 228 patients with NF1 (46.1% male; median age 14 years) were enrolled. Lisch nodules were detected in 82.82% of patients, with highest prevalence during puberty and greater counts in older individuals (*P* < 0.001). Lisch nodules showed a tendency for inferior distribution and likely progressive darkening with age. Choroidal abnormalities were identified in 89.94% of patients, exceeding the prevalence of Lisch nodules (*P* < 0.001). Both the number and area of choroidal abnormalities correlated positively with age (*P* < 0.001), with predominant distribution at the posterior pole. Retinal vascular abnormalities (RVAs), retinal astrocytic hamartomas (RAHs), and iris mammillations were observed in 9.47%, 1.83%, and 8.37% of patients, respectively.

**Conclusions:**

In Asian patients with NF1, older individuals exhibited a greater burden of uveal abnormalities. Lisch nodules demonstrated preliminary evidence of an age-dependent darkening phenotype. Choroidal abnormalities were more prevalent than Lisch nodules and consistently present across age groups, making them valuable diagnostic markers in paediatric patients. Compared with Western cohorts, Chinese paediatric patients with NF1 exhibited earlier onset of both Lisch nodules and choroidal abnormalities. These findings refine ethnicity-specific ocular profiles of NF1 and underscore the importance of timely ophthalmic surveillance, particularly in children.

**Supplementary Information:**

The online version contains supplementary material available at 10.1186/s40662-026-00488-y.

## Background

Neurofibromatosis type 1 (NF1) is an autosomal dominant tumour predisposition syndrome caused by mutations in the *NF1* gene, with an estimated prevalence of 1/4000–1/2000 [1, 2]. The *NF1* gene, located on chromosome 17q11.2, encodes neurofibromin, a protein with a small 300-residue domain that negatively regulates the *RAS* proto-oncogene. Loss of neurofibromin expression leads to increased RAS activity, uncontrolled cell growth, and diverse lesions [3]. NF1 presents with highly variable clinical features, most commonly multiple café-au-lait macules (CALMs) and neurofibromas. Ocular manifestations are central to NF1 diagnosis [4]. Nonetheless, systematic data on ophthalmic findings in Asian populations remain limited, hindering comprehensive characterization and early diagnosis.

Ocular involvement is common in NF1. Lisch nodules (LNs), the most recognized ocular sign, appear as well-defined, dome-shaped elevations on the anterior iris surface [5, 6]. While they rarely impair vision, they are diagnostically significant and were included in the National Institutes of Health (NIH) diagnostic criteria in 1988 [7]. Choroidal abnormalities (CAs), typically asymptomatic, appear as bright patchy nodules on near-infrared reflectance (NIR) imaging with optical coherence tomography (OCT). Due to their high specificity and sensitivity, they were incorporated into the revised diagnostic criteria in 2021 as an alternative ocular sign to LNs [4, 8, 9]. Retinal involvement also occurs in NF1. Retinal vascular abnormalities (RVAs) are increasingly recognized with advances in NIR/OCT imaging [10, 11], though their pathophysiology remains poorly understood. Hyperpigmented spots detected on conventional fundus examination have recently been proposed as a novel ocular marker in NF1 [12]. Less common ocular manifestations include orbital-periorbital plexiform neurofibroma, conjunctival neurofibromas and malignant melanoma, iris mammillations, retinal astrocytic hamartomas (RAHs), and capillary haemangiomas [13–16].

A comprehensive understanding of ocular manifestations is essential for NF1 diagnosis and early detection of vision-threatening lesions. Given the progressive increase in systemic manifestations with age, age-stratified analysis of ocular abnormalities in Asian patients with NF1 is critical for accurate assessment and long-term monitoring.

To address this gap, we conducted a large-scale investigation of NF1-related ocular signs, focusing on uveal and retinal abnormalities in a large Chinese cohort. This study estimated the prevalence of these signs and delineated the clinical ophthalmic spectrum using age-stratified analysis.

## Patients and methods

### Study design

This cross-sectional study was conducted jointly by the Department of Ophthalmology of Xinhua Hospital Affiliated to Shanghai Jiao Tong University School of Medicine, and the Department of Plastic and Reconstructive Surgery of Shanghai Ninth People’s Hospital, Shanghai Jiao Tong University School of Medicine. This study followed the tenets of the Declaration of Helsinki and was approved by the Institutional Review Board of Shanghai Ninth People’s Hospital, Shanghai Jiao Tong University School of Medicine (ID: SH9H-2019-T163-5). All enrolled patients (or parents of the paediatric patients) provided written informed consent.

From July 2024 to October 2024, 231 patients were consecutively recruited; 228 met the revised NIH diagnostic criteria and were included in this study (Figure S1). Demographic data were extracted from electronic medical records. Patients were stratified into five age groups reflecting developmental stages [17–20]: infant and preschool age (0–6 years), school age (7–12 years), puberty (13–18 years), early adulthood (19–30 years; higher gonadal hormone levels), adulthood (31–70 years; declining gonadal hormone levels). This stratification enabled evaluation of age-specific characteristics of NF1-related ocular manifestations.

Comprehensive ophthalmic examinations were performed by experienced ophthalmologists to identify the presence and multidimensional features of NF1-related ocular signs.

### Ophthalmic examinations

A standardized examination protocol was applied to all cooperative patients, including assessment of visual acuity (VA), intraocular pressure (IOP), slit-lamp biomicroscopy, ultra-widefield (UWF) fundus photography, NIR imaging, and OCT (Figure S1). For patients with limited cooperation, all feasible components of the protocol were performed to obtain the most complete dataset.

VA was measured using a standard logarithmic visual acuity chart (GB11533-2011), converted to Snellen VA, and categorized into three subgroups (≤ 20/200, > 20/200 to < 20/40, ≥ 20/40) for statistical analysis. Measurable VA data were obtained from 440 eyes of 220 patients; eight patients were excluded due to poor compliance.

IOP was measured using an iCare ic200 tonometer (iCare Finland Oy). For uncooperative patients, IOP was assessed via digital palpation by an experienced ophthalmologist. Successful IOP measurements were obtained from 451 eyes of 226 patients, except for five eyes of three patients.

Slit-lamp biomicroscopy (TOPCON SL-D301 with DC-4 digital camera) was performed to identify LNs and other anterior segment abnormalities. An experienced ophthalmologist documented the total number of LNs and their distribution across iris quadrants. Pigmentation was classified as lighter (L), equal (E), or darker (D) compared to surrounding iris tissue. Anterior segment photographs were obtained for quality control. A second independent ophthalmologist retrospectively reviewed all images to verify nodule counts. Patients with insufficient image quality were excluded in quantitative analysis. Slit-lamp examination was successfully performed in 227 patients, except for one uncooperative child.

UWF fundus images were acquired using an Optos P200dTx (Optos PLC, Dunfermline, United Kingdom) in 435 eyes of 218 patients to evaluate vitreoretinal abnormalities.

NIR images were obtained using a Spectralis HRA + OCT (Heidelberg Engineering, Heidelberg, Germany) to detect CAs and RVAs. For cooperative patients, five 30° NIR images per eye (posterior pole and four peripheral quadrants) were montaged into a widefield image using Heidelberg Eye Explorer software. CAs were manually delineated, and lesion area was measured using ImageJ software (National Institutes of Health, Bethesda, MD, USA). Each lesion was outlined on the clearest 30° NIR image with reference to the montage to avoid duplication or omission. All measurements were performed by the same ophthalmologist using high-quality images. To assess topographic distribution, the fundus was divided into five zones as described by Nakakura et al. [21], and the occurrence of CAs were documented by zone. OCT was performed using the combined NIR/OCT system of the Spectralis instrument in cooperative patients. NIR images were available for 334 eyes in 169 patients, with corresponding OCT images for 282 eyes in 145 patients.

### Statistical analysis

Continuous variables with normal and skewed distributions were reported as mean ± standard deviation (SD) and median (interquartile range), respectively. Frequencies and proportions were reported for categorical variables. Prevalence of LNs and CAs across age groups was compared using chi-square and Fisher’s exact tests. Chi-square and McNemar’s tests were used to compare prevalence between sexes and between right and left eyes, respectively. McNemar’s test was also applied to patients who underwent both examinations.

Spearman’s ρ was calculated to assess correlations between age, number of LNs, number of CAs, and area of CAs. The paired Wilcoxon rank-sum test was used to analyse interocular differences in the number of LNs, number of CAs, and area of CAs.

To evaluate spatial distribution of LNs, we constructed a negative binomial mixed model with nodule count per quadrant as the dependent variable and quadrant as the fixed effect. A nested design with laterality and patient IDs being the inner and outer level was adopted for the random intercept to account for within-patient correlations (both eyes of the same patient). Pairwise comparisons between quadrants were based on estimated marginal means, with Bonferroni adjustment for *P* values.

Fisher’s exact test was used to compare pigmentation categories of LNs across age groups. Prevalence of CAs across fundus regions was compared using chi-square tests. The number of affected regions across age groups was analysed using the Wilcoxon rank-sum test.

All tests were two-tailed, with statistical significance set at *P* < 0.05. The Benjamini–Hochberg method was applied to adjust for multiple comparisons in pairwise analyses. All analyses and visualizations were performed using R software (version 4.2.2).

## Results

### Demographic and clinical characteristics

In total, 228 Asian patients with NF1 were included in the analysis (Table 1). The cohort comprised 105 males (46.1%) and 123 females (53.9%), with a median age of 14 years (range: 3–67 years) at ophthalmic assessment. VA was ≥ 20/40 in 80.5% of eyes. Poor VA was observed in 16 eyes of 15 patients, prompting recommendations for further ophthalmic evaluation and magnetic resonance imaging. IOP was within the normal range in 92.5% of eyes.

**Table 1:** Demographic and clinical characteristics of the cohort

Patients		
		N = 228
Sex, n (%)	Male	105 (46.1)
	Female	123 (53.9)
Age (years), median (IQR)		14 (17.25)
Age group, n (%)	0–6 years	31 (13.6)
	7–12 years	67 (29.4)
	13–18 years	37 (16.2)
	19–30 years	50 (21.9)
	31–70 years	43 (18.9)

Eyes		
		N = 456
VA group, n (%)	20/200 or worse	16 (3.6) *
	better than 20/200 but worse than 20/40	70 (15.9) *
	20/40 or better	354 (80.5) *
IOP group, n (%)	< 10 mmHg	21 (4.6) †
	10–21 mmHg	359 (79.6) †
	> 21 mmHg	13 (2.9) †
	Tn	58 (12.9) †

*IQR* = interquartile range; *VA* = visual acuity; *IOP* = intraocular pressure; *Tn* = normal IOP via digital palpation; *, VA results were available in 440 eyes of 220 patients; †, IOP results were available in 451 eyes of 226 patients

### Lisch nodules

LNs were detected in 188 of 227 patients (82.82%; 95% confidence interval [CI]: 77.27%–87.49%) who underwent slit-lamp examination. Among these, 164 patients (72.25%) had bilateral involvement, while 21 had unilateral nodules. In three patients with monocular corneal opacity, LNs were detected in the fellow eye. At the eye level, Lisch nodules were observed in 352 of 451 assessable eyes, corresponding to a prevalence of 78.05%.

**Fig. 1:**
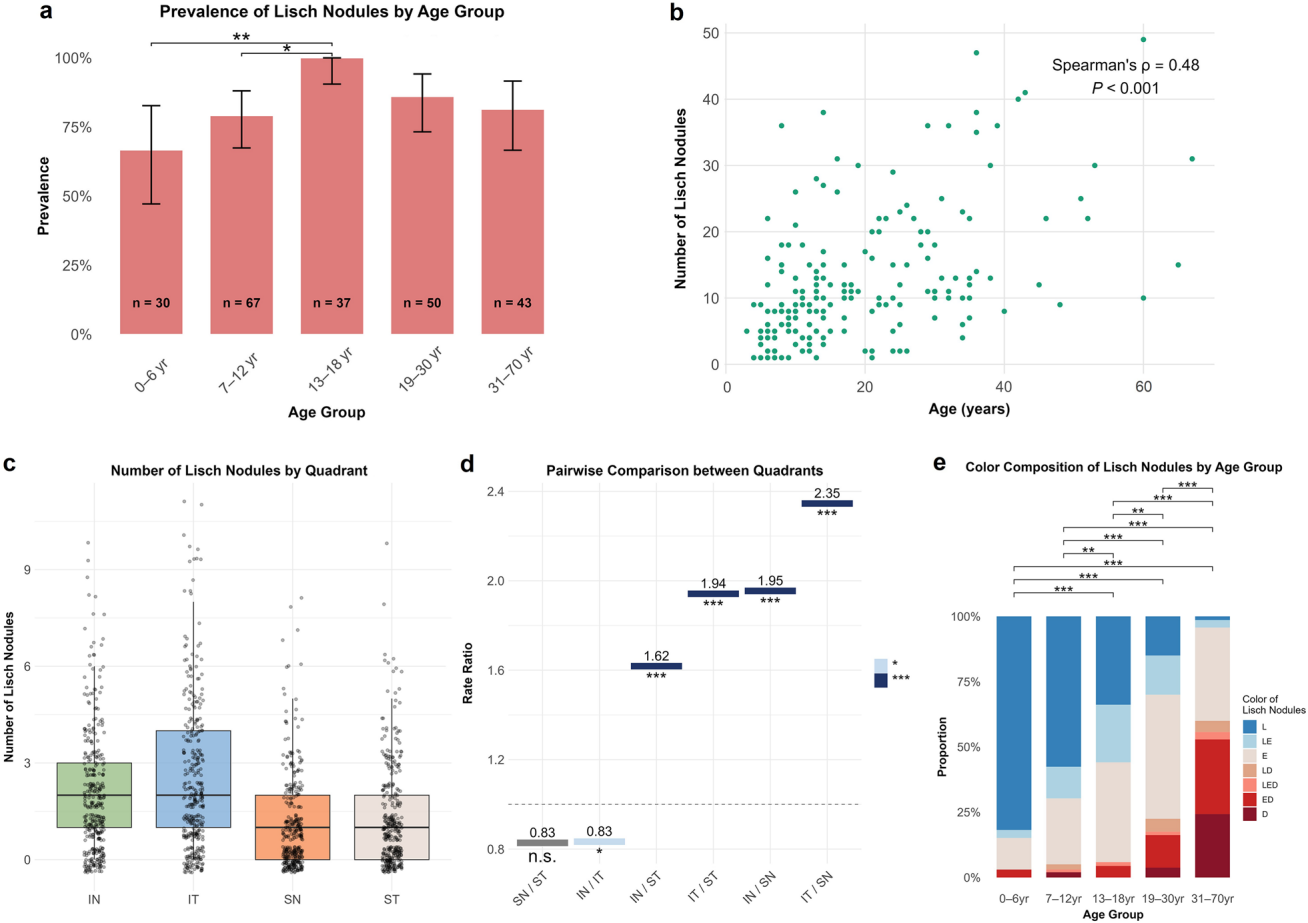
Multidimensional phenotypic profile of Lisch nodules (LNs) in Chinese patients with neurofibromatosis type 1. **a** Prevalence of LNs across age groups. 66.67%, 79.10%, 100.00%, 86.00% and 81.40% for 0–6 year, 7–12 year, 13–18 year, 19–30 year and 31–70 year age groups, respectively. Chi-square test with Benjamini–Hochberg correction used for multiple comparisons. **b** Total number of LNs positively correlated with age (Spearman’s ρ = 0.48, *P* < 0.001). **c** Distribution of LNs by quadrant. Inferior nasal (median: 2, interquartile range [IQR]: 2) and inferior temporal (median: 2, IQR: 3) quadrants contained more nodules than superior nasal (median: 1, IQR: 2) and superior temporal (median: 1, IQR: 2). **d** Pairwise comparisons between quadrants based on estimated marginal means. Bonferroni correction was applied. Each bar and number above it represent the estimated rate ratio. Bar colour and asterisks indicate statistical significance. **e** Colour composition of LNs by age group. Significant differences were observed across age groups. Lighter nodules predominated in younger patients, while darker nodules were more frequent in older patients. Fisher’s exact test with Benjamini–Hochberg correction used for multiple pairwise comparisons. *P < 0.05, ** *P* < 0.01, *** *P* < 0.001. n.s., not significant; IN, inferior nasal; IT, inferior temporal; SN, superior nasal; ST, superior temporal. L, lighter than iris; E, equal to iris; D, darker than iris; LE, both lighter than iris and equal to iris; ED, both equal to iris and darker than iris; LD, both lighter than iris and darker than iris; LED, lighter than iris, equal to iris and darker than iris

Prevalence of LNs was not significantly associated with patient sex (*P* = 0.629) or laterality (*P* = 0.663). However, prevalence peaked in adolescents aged 13–18 years, significantly higher than that in children aged 0–6 years (*P* < 0.01) and 7–12 years (*P* < 0.05) (Fig. 1a, Table 2). Adults showed slightly lower prevalence compared to adolescents, but rates remained higher than in younger groups, without statistical significance.

**Table 2:** Prevalence and quantitative burden of Lisch nodules and choroidal abnormalities in Chinese patients with neurofibromatosis type 1

Prevalence of Lisch nodules and choroidal abnormalities									
	Overall	0–6 years		7–12 years	13–18 years		19–30 years		31–70 years
Lisch nodules, n (%)^a^	188 (82.82%)	20 (66.67%)		53 (79.10%)	37 (100.0%)		43 (86.00%)		35 (81.40%)
Choroidal abnormalities, n (%)^b^	152 (89.94%)	16 (88.89%)		48 (87.27%)	30 (100.0%)		30 (90.91%)		28 (84.85%)

Quantitative burden of Lisch nodules and choroidal abnormalities									
			Median			IQR		Range	
Number of Lisch nodules (OU)^c^			10			10.25		1–49	
Number of choroidal abnormalities (OU)^d^			28			27.5		1–68	
Area of choroidal abnormalities (OU)^d^ (mm^2^)			44.32			46.13		3.10–181.58	

*OU* = *oculus uterque* (both eyes); *IQR* = interquartile range

^a^ Slit-lamp examination conducted in 227 patients; ^b^ NIR images available for 169 patients; ^c^ Data available for 184 patients; ^d^ Data available for 123 patients

**Fig. 2:**
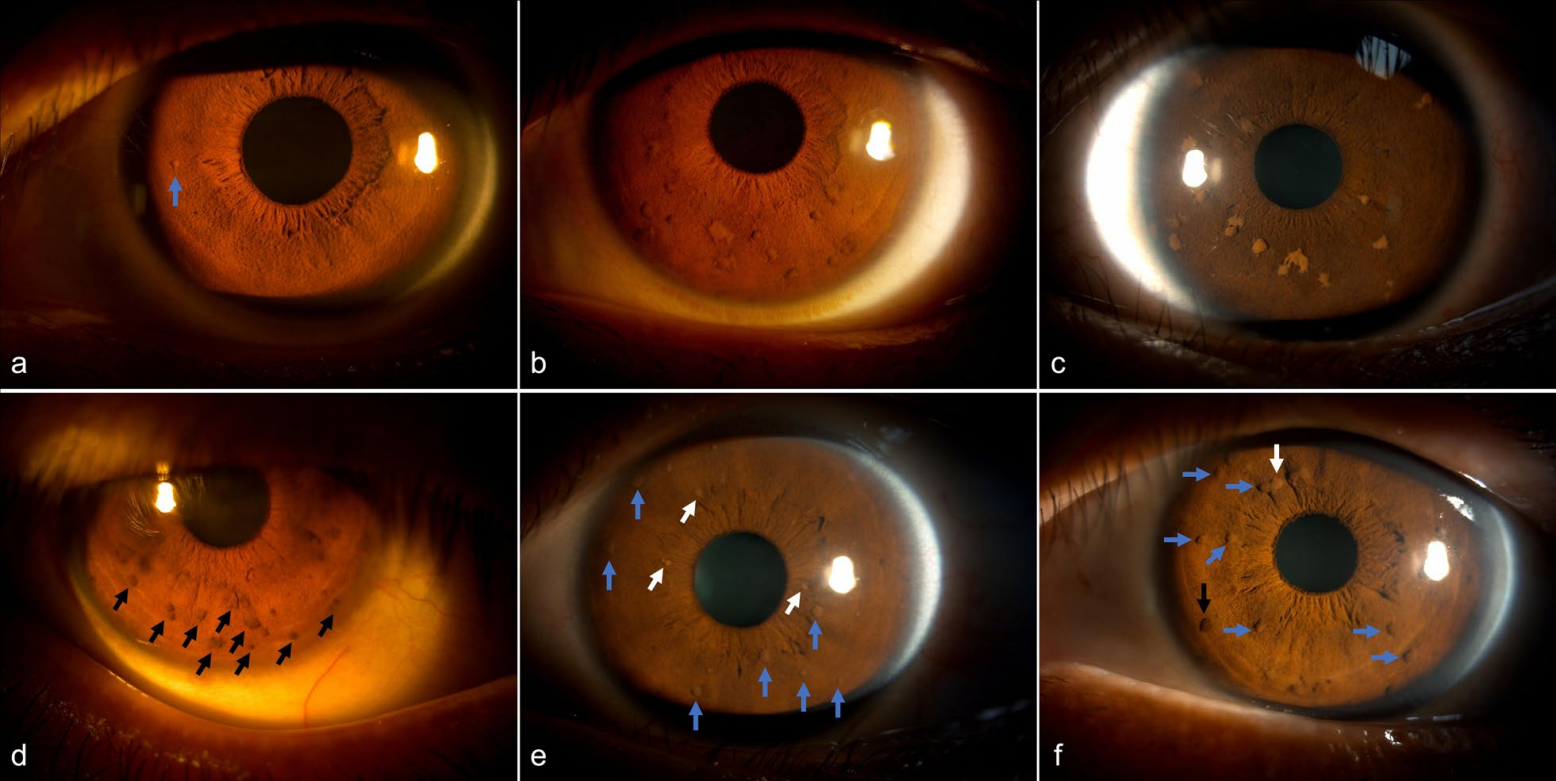
Representative images of Lisch nodules in Chinese patients with neurofibromatosis type 1. **a** Unilateral solitary nodule (arrow). **b**–**d** Multiple nodules with pigmentation equal to (**b**), lighter (**c**), or darker (**d**) than the iris. **e**, **f** Heterogeneous pigmentation within multiple nodules: equal (blue arrows), lighter (white arrows), and darker (black arrow) compared to the background iris

Of the 188 patients with LNs, nine (4.79%) exhibited a solitary nodule (Fig. 2a; mean age: 8.6 years). The remainder had multiple nodules, fulfilling NF1 diagnostic criteria. In 184 patients with assessable data (excluding three with unilateral corneal opacity and one with poor image quality), the median total number of bilateral nodules was 10 (Table 2). A significant positive correlation was found between nodule count and age (Spearman’s ρ = 0.48, *P* < 0.001) (Fig. 1b). No significant interocular difference in nodule number was observed (*P* = 0.635) (Figure S2).

To investigate spatial distribution, the number of LNs in each iris quadrant was analysed (Fig. 1c, d). After excluding 24 patients with unilateral data and one with poor bilateral image quality, 350 eyes from 187 patients were included. Nodule counts varied significantly across quadrants. Pairwise comparisons confirmed a consistent predilection for inferior quadrants compared with superior quadrants. Rate ratios (RRs) demonstrated substantially higher counts in inferior quadrants: inferior nasal vs. superior nasal (RR = 1.95, *P* < 0.001), inferior nasal vs. superior temporal (RR = 1.62, *P* < 0.001), inferior temporal vs. superior nasal (RR = 2.35, *P* < 0.001), and inferior temporal vs. superior temporal (RR = 1.94, *P* < 0.001) (Fig. 1d, Table S1). Within inferior quadrants, a subtle difference was observed (RR = 0.83, *P* = 0.014), though this was not clinically significant. Comparisons within superior quadrants showed no statistically significant differences (RR = 0.83, *P* = 0.090) (Fig. 1d, Table S1).

The colour of LNs was assessed relative to adjacent iris tissue for the first time in an Asian population. LNs were lighter, equal, or darker than the surrounding iris in 34.29%, 33.71%, and 6.29% of eyes, respectively (Fig. 2b–d). The remaining eyes exhibited LNs with different colours (Fig. 2e, f). A distinctive age-related shift in pigmentation was observed. In patients aged 0–6 years, 81.82% of eyes had lighter nodules, whereas only 1.43% of eyes in patients aged 31–70 years showed exclusively lighter nodules. Darker nodules were rare in younger children but predominantly present in 24.29% of eyes in the 31–70 year age group. Overall, darker nodules were more common in older patients, while lighter nodules predominated in younger groups (Fig. 1e).

### Choroidal abnormalities

**Fig. 3:**
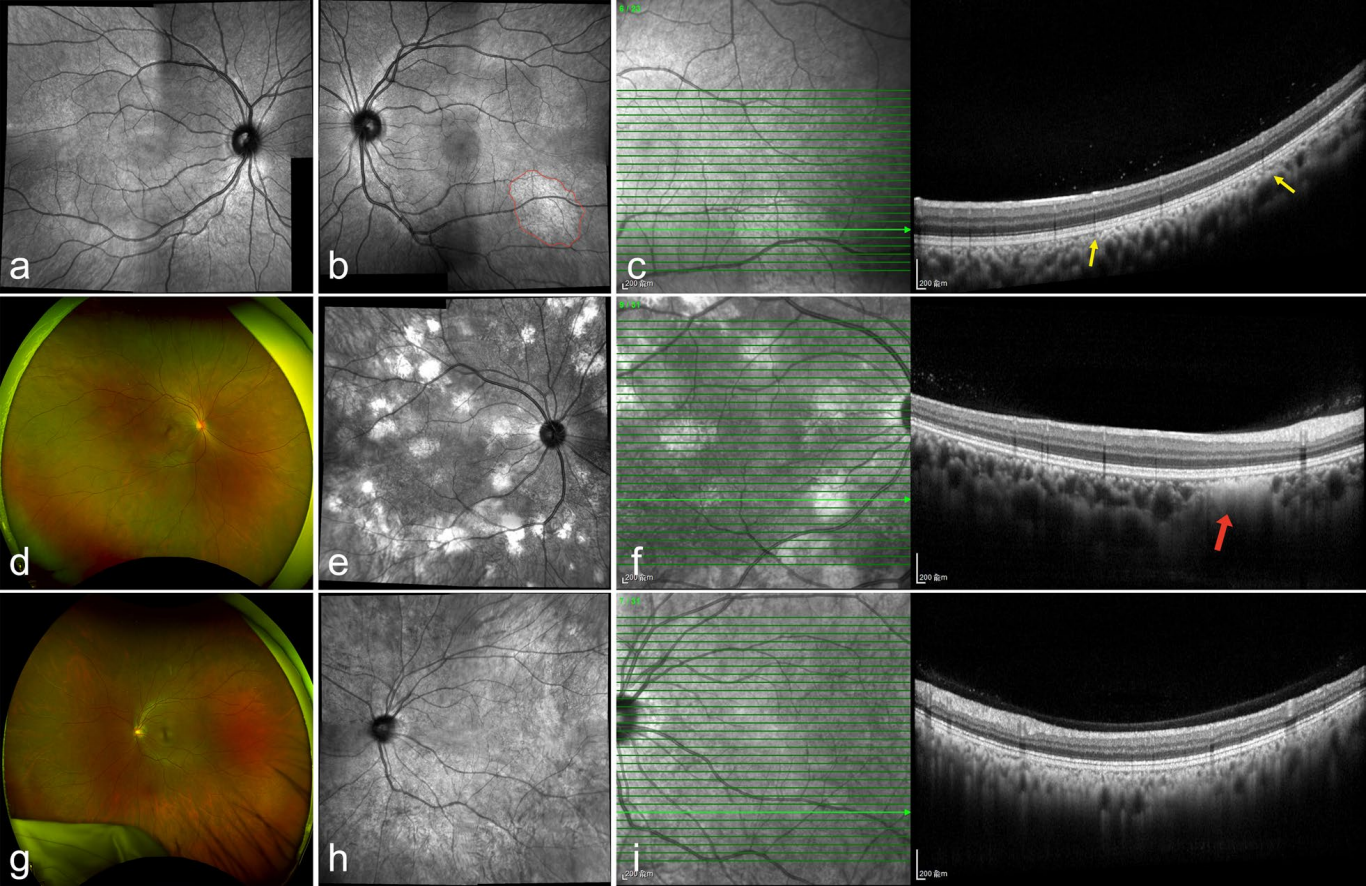
Representative multimodal imaging of choroidal abnormalities (CAs) in Chinese patients with neurofibromatosis type 1. **a**–**c** Unilateral, solitary CA in the left eye of one patient. **a** Montage near-infrared reflectance (NIR) image of the unaffected right eye. **b** Montage NIR image of the left eye shows a single, bright patchy lesion (red outline) inferotemporal to the macula. **c** Corresponding optical coherence tomography (OCT) reveals an irregular hyperreflective lesion interwoven with choroidal vasculature (yellow arrows), consistent with a placoid nodule. **d**–**f** Multiple discrete CAs in the right eye. **d** Ultra-widefield (UWF) fundus image appears unremarkable. **e** Montage NIR image shows multiple well-defined bright nodules. **f** OCT demonstrates a dome-shaped hyperreflective mass (red arrow) compressing choroidal vessels. **g**–**i** Diffuse CAs in the left eye of a 39-year-old female. **g** UWF fundus image appears unremarkable. **h** Montage NIR image shows extensive diffuse hyperreflective change. **i** OCT reveals extensive hyperreflective lesions interwoven with compressed choroidal vasculature, indicative of diffuse abnormalities. The fellow eye of this patient showed multiple dome-shaped nodules similar to (**d**–**f**)

**Fig. 4:**
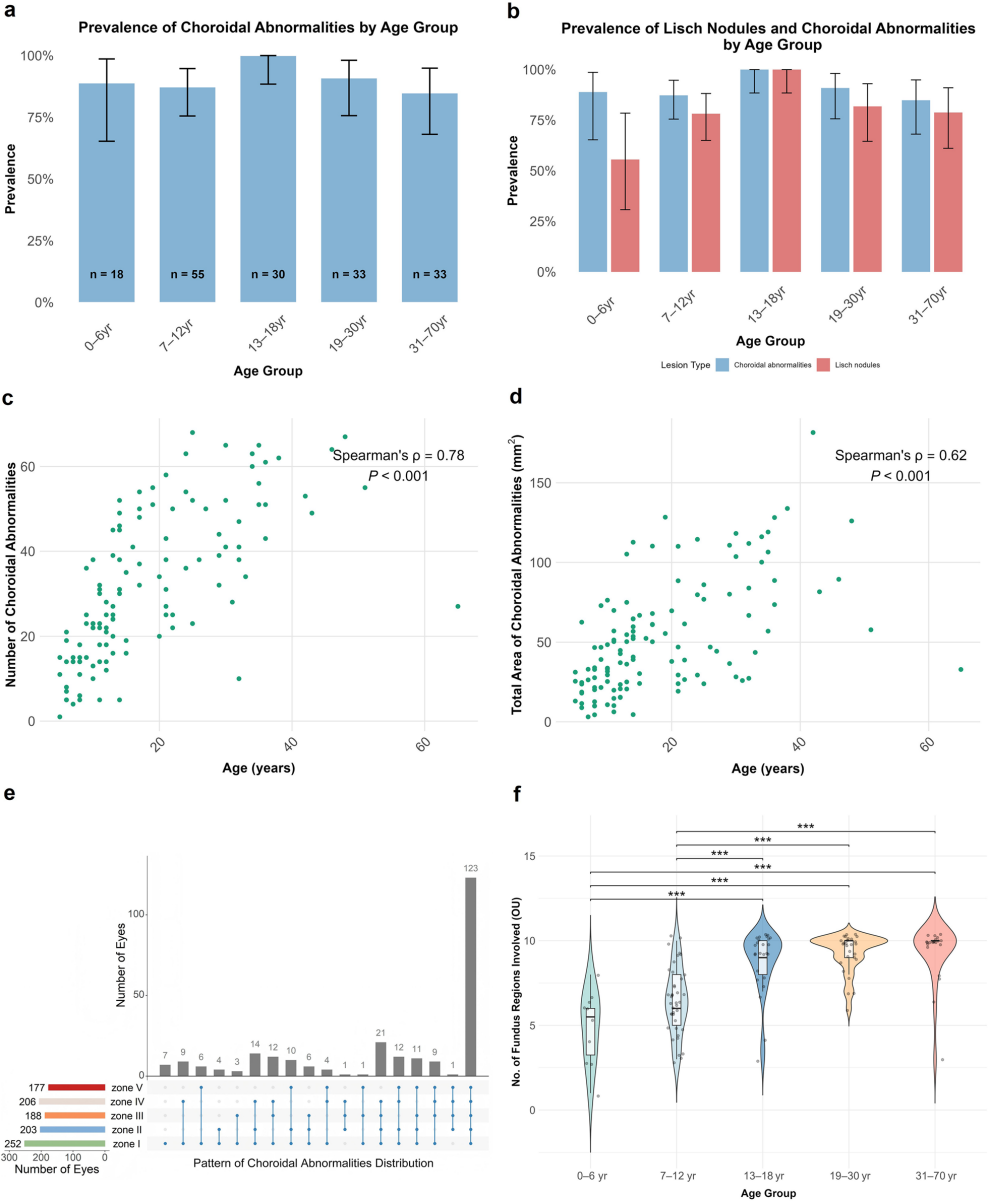
Multidimensional phenotypic profile of choroidal abnormalities (CAs) in Chinese patients with neurofibromatosis type 1. **a** Prevalence of CAs across age groups. All age groups exhibited consistently high prevalence (0–6 years: 88.89%; 7–12 years: 87.27%; 13–18 years: 100%; 19–30 years: 90.91%; 31–70 years: 84.85%), with no significant differences (Fisher’s exact test, *P* = 0.215). **b** Prevalence of Lisch nodules versus CAs in patients who underwent both examinations (n = 169). **c** Correlation between total number of CAs and age. The total number of CAs positively corelated with age (Spearman’s ρ = 0.78, *P* < 0.001). **d** Correlation between total area of CAs and age. The total area of CAs also showed a significant positive correlation with age (Spearman’s ρ = 0.62, *P* < 0.001). **e** Spatial distribution of CAs. Upset plot illustrating involvement of fundus regions. Most eyes had multiple regions affected; only seven eyes showed abnormalities restricted to the posterior pole. All five regions were involved in 123 of 254 eyes. **f** Number of fundus regions affected across age groups. Younger groups (0–6 years, 7–12 years) had significantly fewer regions involved compared with older groups (Wilcoxon rank-sum test with Benjamini–Hochberg correction, ***P < 0.001)

Among the 169 patients with NF1 who underwent NIR fundus photography, CAs (Fig. 3) were identified in 152 (89.94%; 95% CI: 84.38%–94.03%) and 142 (84.02%) patients exhibited bilateral involvement. Unilateral CAs were identified in six patients. Four patients had only monocular data due to corneal opacity (two cases) or retinal detachment (two cases). Prevalence was comparable across age groups (*P* = 0.215) (Fig. 4a, Table 2) and showed no significant association with sex (*P* > 0.999) or laterality (*P* = 0.683).

In patients who underwent both slit-lamp examination and NIR imaging (n = 169), CAs were significantly more prevalent than LNs (*P* < 0.001) (Table S2). Subgroup analysis showed borderline significance in the 0–6 year age group (*P* = 0.041, Benjamini–Hochberg adjusted *P* = 0.147) (Fig. 4b).

Most patients had multiple lesions, except for two paediatric cases: one boy with a single lesion detected on montage widefield NIR imaging of both eyes (Fig. 3a–c), and one girl with imaging limited to the left eye due to poor cooperation. In 123 patients with high-quality NIR images, the median number of binocular lesions was 28 (Table 2). The total number of lesions correlated positively with age (Spearman’s ρ = 0.78, *P* < 0.001) (Fig. 4c). No significant interocular differences were observed (*P* = 0.721; Figure S3). Similarly, the total lesion area correlated positively with age (Spearman’s ρ = 0.62, *P* < 0.001) (Fig. 4d), with no significant interocular difference (*P* = 0.324; Figure S4).

CA distribution was documented in 254 eyes of 136 patients with confirmed choroidal involvement. CAs exhibited a significant predilection for the posterior pole (zone I) compared to other fundus regions (*P* < 0.001), followed by the superior nasal (zone IV) and superior temporal (zone II) regions (Fig. 4e, Figure S5). Two eyes from two distinct patients demonstrated posterior pole-sparing pattern of CAs. CAs involved all five fundus regions in 123 of 254 eyes (48.43%), while seven eyes showed lesions restricted to the posterior pole (Fig. 4e). Stratified analysis revealed a positive correlation between the number of affected regions and advancing age (Fig. 4f).

### Retinal vascular abnormalities and other fundus findings

**Fig. 5:**
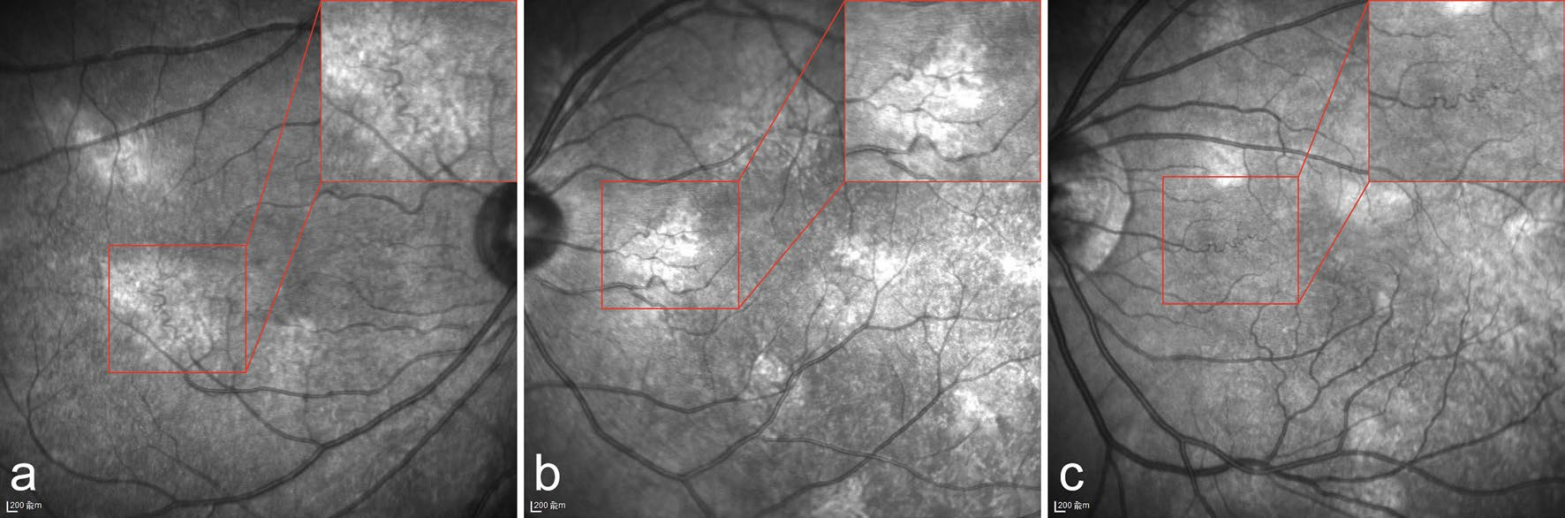
Typical retinal vascular abnormalities (RVAs) in Chinese patients with neurofibromatosis type 1. **a**, **b** Near-infrared reflectance (NIR) images show the abnormal tortuous retinal vessels originating from branch of the retinal venules (**a**) and the optic disc (**b**), running over choroidal abnormalities. **c** NIR image shows tortuous retinal vessel originating from the optic disc without clear topographic correspondence with choroidal abnormalities

Based on NIR imaging, RVAs were identified in 16 of 169 patients (9.47%; 95% CI: 5.51%–14.92%); all cases were unilateral. In eight eyes, tortuous retinal vessels overlaid regions of CAs (Fig. 5a, b), while in the other eight, no obvious topographic correspondence with CAs was observed (Fig. 5c).

**Fig. 6:**
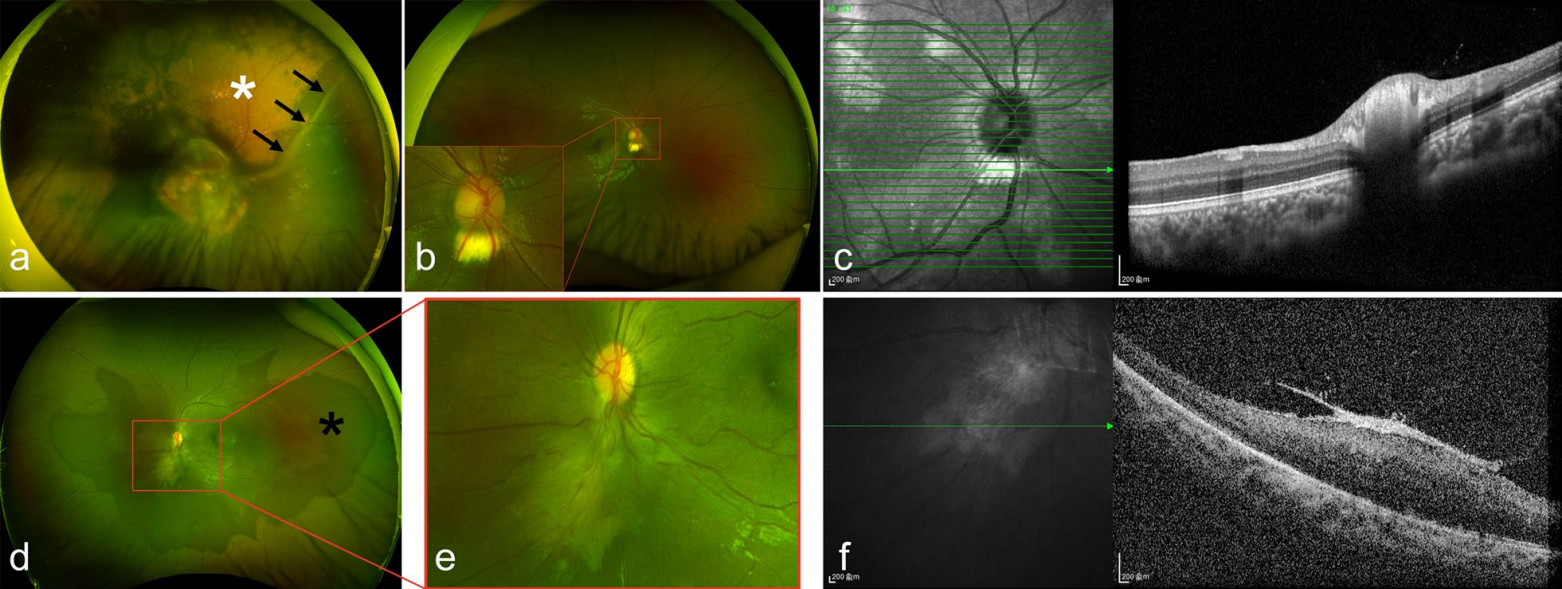
Retinal astrocytic hamartomas (RAHs) and other fundus abnormalities in Chinese patients with neurofibromatosis type 1. **a** Ultra-widefield (UWF) fundus image shows a large RAH in the posterior pole with secondary inferior retinal detachment (black arrows) and extensive exudation (white asterisk). **b** UWF fundus image shows a yellowish elevated RAH beneath the optic disc. Enlarged view of boxed area shown below. **c** Optical coherence tomography (OCT) image reveals a dome-shaped hyperreflective mass within the retinal nerve fibre layer, consistent with RAH. **d** UWF fundus image shows an epiretinal membrane beneath the optic disc with localized dark without pressure degeneration (black asterisk). **e** Enlarged view of boxed area in (**d**) shows retinal contraction caused by the epiretinal membrane. **f** OCT image demonstrates local retinal thickening caused by contraction of epiretinal membrane

Among 218 patients who underwent UWF fundus photography, RAHs were identified in five eyes of four patients (1.83%; 95% CI: 0.50%–4.63%). Except for one eye complicated by secondary retinal detachment (Fig. 6a), RAHs appeared as round, yellowish-elevated lesions adjacent to the optic disc on UWF images (Fig. 6b), and as dome-shaped hyper-reflective masses within the retinal nerve fibre layer with inner retinal disorganization on OCT (Fig. 6c). A bilateral epiretinal membrane (ERM) was noted in one patient (Fig. 6d–f). Additionally, total retinal detachment secondary to Coats disease was observed in one eye. No hyperpigmented spots were detected in this cohort.

### Other anterior segmental abnormalities

**Fig. 7:**
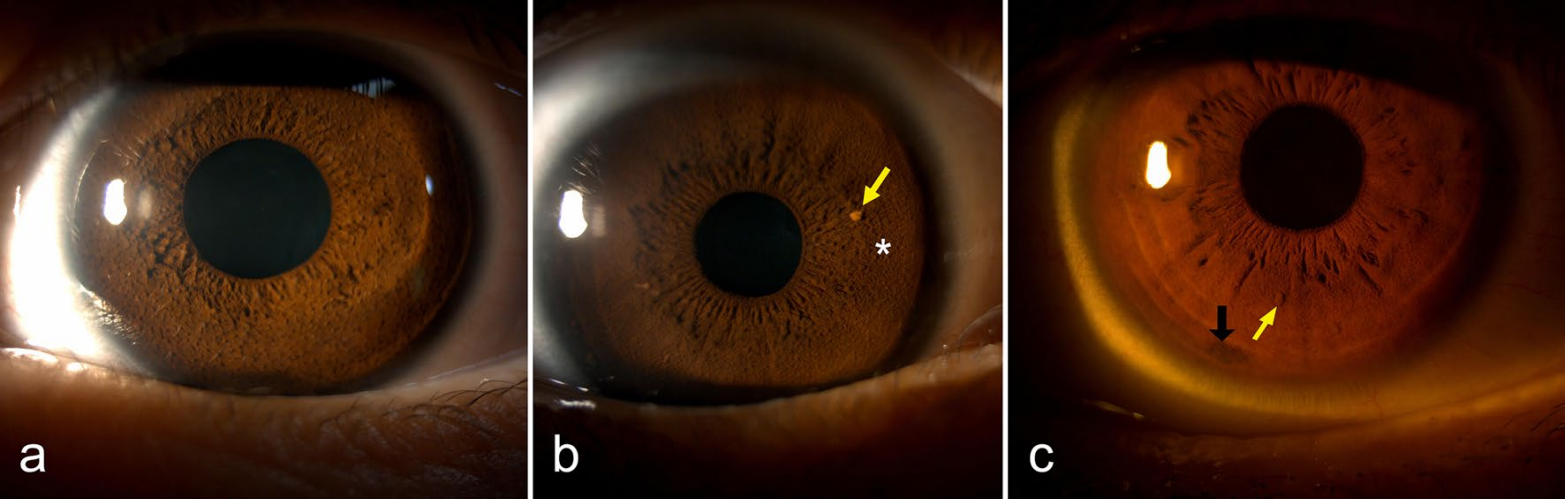
Iris mammillations and iris naevus in Chinese patients with neurofibromatosis type 1. **a** Diffuse conical villiform elevations on the iris surface, suggesting iris mammillations, was noted. **b** Iris mammillations (asterisk) was observed beneath a solitary Lisch nodule (yellow arrow). **c** Iris naevus (black arrow) and Lisch nodule (yellow arrow) were noted in the same eye

Iris mammillations (Fig. 7a, b), presenting as regularly arranged conical villiform elevations on the iris surface and leading to a bumpy iris appearance, were observed in 30 eyes of 19 patients (8.37%; 95% CI: 5.11%–12.76%). Of these, 22 eyes of 17 patients also had typical LNs (Fig. 7b). Iris naevi (Fig. 7c) were detected in 66 eyes of 53 patients (23.35%; 95% CI: 18.01%–29.40%). Severe corneal degeneration was noted in three eyes of three patients, likely due to long-term eyelid coverage by neurofibromas in two cases and advanced Coats disease in one case.

## Discussion

This cross-sectional study comprehensively characterized uveal and retinal manifestations in a large Asian NF1 cohort, providing new insights into age-related ocular profiles in this population.

VA measurements were available for 440 eyes of 220 patients, with acceptable VA in 80.5% and poor VA in 16 eyes. After preliminary autorefraction correction, seven eyes (from seven patients) had persistently poor VA due to corneal opacity (two eyes), retinal detachment secondary to RAH (one eye) or Coats disease (one eye), giant facial neurofibroma (one eye), and prior trauma (two eyes). Most eyes achieved corrected VA of 20/25 or better. However, previous electrophysiological studies have demonstrated subclinical neuroretinal and optic pathway dysfunction in patients with NF1 with best-corrected VA (BCVA) of 20/20 or better and no optic pathway gliomas, suggesting that BCVA alone may underestimate visual impairment in NF1 [22, 23]. Future studies incorporating electrophysiological testing are warranted to fully assess visual function in NF1.

IOP was successfully measured in 451 eyes of 226 patients. Thirteen eyes showed elevated IOP, attributed to ocular compression during manual eyelid retraction by patients during examination. Subsequent digital palpation confirmed normal IOP in all affected eyes.

LNs were observed in 82.82% of patients with NF1 in this study, consistent with previous reports [5, 24–27]. A slightly lower prevalence in adults compared with adolescents was noted, which appears inconsistent with the established understanding that prevalence increases with age [13, 28, 29]. Patients with mosaic NF1 without LNs were presumed to have partly contributed to this difference. After excluding patients with mosaic NF1, the prevalence in these two adult-age groups was closer to that in the 13–18 year group (Figure S6). These findings suggest that the prevalence of Lisch nodules may plateau after puberty. Furthermore, a higher prevalence of Lisch nodules in paediatric patients with NF1 (82.09%) was observed compared with prior studies reporting 41%–62.8% [24, 30, 31]. This may indicate earlier onset of LNs in the Chinese NF1 population.

A previous study reported darker LNs relative to the surrounding iris in an adult Chinese patient [32]. In this study, we classified Lisch nodule colour relative to the surrounding iris tissue and analysed the colour composition of LNs across different age groups for the first time. We observed that eyes exhibiting darker Lisch nodules were more common in older age groups. While Lisch nodules are established melanocytic hamartomas, recent work has highlighted histologic heterogeneity [5, 6, 33–35]. We speculate that initial extracellular matrix and melanocyte accumulation presents as lighter LNs, with subsequent melanin deposition leading to gradual darkening. Given the cross-sectional design, longitudinal studies are needed to confirm the evolution of Lisch nodule pigmentation with extended follow‑up. Dark iris pigmentation may reduce detectability in Asian populations. Because of their early onset and subtle coloration, examination of Lisch nodules should be prioritized in young patients with NF1 to improve early detection.

In this cohort, LNs showed a predilection for the inferior iris, consistent with studies in Caucasian populations attributing this to differential ultraviolet light exposure between superior and inferior hemifields [26, 36, 37]. The absence of inter-racial differences suggests this asymmetric distribution is a universal characteristic, underscoring the priority of inferior quadrants during clinical assessment.

CAs were detected in 89.94% of patients, consistent with previously reported prevalence in adult NF1 populations (82%–100%) [38]. Nonetheless, the prevalence in paediatric patients was higher than that reported in prior paediatric NF1 studies [30, 31, 39–43], suggesting earlier onset and possibly greater susceptibility in the Chinese population, which is analogous to the pattern reported for LNs.

In patients who underwent both slit-lamp examination and NIR imaging, CAs were significantly more prevalent than LNs, indicating they may serve as more sensitive diagnostic markers than Lisch nodules in Chinese patients with NF1, particularly in the paediatric subgroup. Although subgroup analysis showed only borderline significance in patients aged 0–6 years (McNemar’s test, *P* = 0.041; Benjamini–Hochberg adjusted* P* = 0.147), likely due to limited sample size, CAs remain valuable ophthalmic biomarkers in paediatric NF1. Given their predominance at the posterior pole, a single NIR image focused on this region may be more feasible than slit-lamp examination in uncooperative children.

CAs were observed across all fundus regions but predominantly at the posterior pole. This is consistent with prior studies and may reflect greater choroidal thickness and melanocyte density in this region [8, 21, 40]. Notably, the region superior‑nasal to the optic disc (zone IV) demonstrated a high prevalence of CAs, second only to the posterior pole, contrasting with previous reports. This suggests zone IV may represent another frequent site of involvement in Chinese patients and merits specific attention during screening. Involvement of more fundus regions was noted in older patients, suggesting expansion of CAs with age, which requires further longitudinal confirmation.

Building on the two morphological subtypes of CAs proposed by Abdolrahimzadeh et al. [44], we identified two additional atypical variants in our cohort. The first appeared as a well-defined hyperreflective area on NIR imaging, corresponding to a placoid hyperreflective lesion interwoven with choroidal vasculature on OCT (Fig. 3b, c). The second presented as an extensive, ill-defined hyperreflective change on NIR, with OCT showing a prominent hyperreflective mass compressing the underlying choroidal vessels (Fig. 3h, i). We speculate the former may represent an early form of CA, whereas the latter corresponds to an advanced confluent stage (diffuse CAs). These findings expand the clinical spectrum and may reduce misdiagnosis or under‑detection in practice. Recent histological studies confirmed choroidal abnormalities as aggregates of melanocytes with heterogeneous morphology, corresponding to both Abdolrahimzadeh’s subtypes, and different depths within the choroid [35, 44].

Moramarco et al. reported hyperpigmented spots on fundus examination as a new ocular sign of NF1 [12]. Given the anatomical overlap between these hyperpigmented spots and CAs, along with confirmation via red-only laser Optos imaging, these were considered manifestations of the same lesion type [12]. However, no hyperpigmented spots were observed in this cohort, possibly due to differences in retinal pigment epithelium pigmentation or histological heterogeneity of CAs between the two study populations [35].

In this cohort, RVAs were detected in 9.47% of patients with NF1, with a mean age of 29.88 ± 15.72 years. While three morphological patterns have been previously described—simple vascular tortuosity, corkscrew retinal vessels, and moyamoya-like configuration [45]—all cases in this study exhibited only simple tortuosity (Fig. 5). Progression of RVAs from simple to corkscrew patterns has been reported in paediatric patients with NF1 [46, 47], suggesting RVAs evolve over time. The relatively older age and exclusive presence of simple tortuosity in this cohort suggest later onset and/or slower progression in Chinese patients. Further longitudinal studies are warranted to clarify the natural history of RVAs in Asian NF1 populations.

Although the exact pathogenesis of RVAs remains unclear, several hypotheses have been proposed. Abdolrahimzadeh et al. reported six patients with RVAs overlying CAs and suggested dysfunction of neural crest-derived vasomotor cells [48]. Cassiman et al. observed a similar phenomenon but hypothesized that angiogenic factors from choroidal lesions might alter retinal vasculature [49]. Another proposal involved mechanical compression of choroidal capillaries by choroidal nodules leading to retinal vascular changes [50]. In our cohort, RVAs were located over CAs in half of the RVA-positive cases, while the remainder showed no clear topographic correlation, suggesting the spatial relationship may be coincidental. Existing hypotheses may not fully explain RVA pathogenesis. Given the relatively low prevalence of RVAs in this study, the potential role of choroidal abnormalities in RVA development warrants further investigation.

RAHs, typical retinal hamartomas commonly observed in tuberous sclerosis complex [51], have also been reported to occur in patients with NF1 in a limited number of case reports [14, 52]. Here, we report the detection of RAHs in 1.83% patients with NF1 who underwent UWF fundus photography. Except for one eye complicated by secondary retinal detachment, the remaining four eyes with RAHs were stable, reflecting the slow-growing nature of NF1-related RAHs, similar to those seen in tuberous sclerosis complex [51, 53].

The ERM, commonly reported in neurofibromatosis type 2 (NF2) [54–58], was observed in both eyes of a six-year-old girl in this cohort. She met five of the seven NIH diagnostic criteria for NF1 (multiple CALMs, axillary freckling, neurofibromas, multiple CAs, and a pathogenic *NF1* gene mutation) and had no other systemic conditions, confirming NF1 and excluding NF2. The ERM in this patient exhibited distinct morphological features compared with typical NF2-associated ERM. Both ERMs were located inferior to the optic disc, causing localized retinal traction, while the macular region remained largely unaffected. In contrast, NF2-associated ERM typically involves the macula, with characteristic features including anteriorly projecting edges into the vitreous despite incomplete posterior vitreous detachment (flame-shaped appearance), absence of cystoid macular edema, and irregular or partially absent internal limiting membrane [54, 56, 58]. Given the limitation of a single case, further accumulation of cases is needed to clarify ERM morphology in NF1 and distinguish it from NF2-associated ERM.

Iris mammillations, first described by Coats in 1912, are a common differential diagnosis of LNs [59]. Although primarily distinguished by morphology, the comorbidities of these two entities have been reported in patients with NF1 [15]. Nonetheless, the prevalence and clinical features of iris mammillations have not yet been systematically studied in a large NF1 cohort. In this Chinese cohort, iris mammillations had an estimated prevalence of 8.37%, with nearly 90% of affected individuals also exhibiting LNs. This high co-occurrence suggests that iris mammillations and LNs may represent different morphological manifestations of a shared pathological process. The underlying pathogenesis and precise relationship between these two findings remain to be elucidated.

## Limitations

This study had several limitations. First, as this was a cross-sectional analysis, the natural progression of uveal manifestations in patients with NF1 could not be definitively established. Although age-stratified analyses revealed age-related differences in the multidimensional characteristics of uveal manifestations, these findings did not confirm disease progression over time. Longitudinal follow-up studies, which are currently underway, are required to validate these observations. Second, hospital-based recruitment may have introduced selection bias. Additionally, more than half of the enrolled patients lacked genetic testing results. Because pathogenic *NF1* mutations are a diagnostic criterion, absence of genetic data may have excluded potential NF1 cases. Although only three participants were excluded, this may have introduced subtle cohort bias. Third, uncorrected VA was reported in this study. Although preliminary autorefraction-corrected VA indicated that most impairments stemmed from refractive errors, the lack of BCVA data limits accurate assessment of visual function and precludes further exploration of the impact of NF1-related ocular findings on visual outcomes.

## Conclusions

This cross-sectional study characterised the uveal and retinal manifestations of NF1 in a large Chinese cohort, providing novel insights into Asian patients with NF1. A greater burden of uveal abnormalities was observed in older patients. LNs, detected in 82.82% of the patients, demonstrated preliminary evidence of an age-dependent darkening phenotype and a consistent inferior iris predilection. CAs were more prevalent than LNs, with stable prevalence across all age groups and a predominant distribution at the posterior pole, rendering them particularly valuable diagnostic indicators in paediatric patients with NF1. Additionally, Chinese paediatric patients exhibited earlier onset of both LNs and CAs compared to previous Western cohorts.

Collectively, these findings refine the ethnicity-specific ocular characteristics of NF1, underscore the necessity for timely and regular ophthalmic surveillance in NF1, and warrant further longitudinal investigation into the natural history of NF1-related ophthalmic pathology.

## Supplementary Information

Supplementary Material 1.

## Data Availability

The data supporting the findings of this study are available from the corresponding author upon request.

## Abbreviations

BCVA: Best-corrected visual acuity
CAs: Choroidal abnormalities
CALMs: Café-au-lait macules
CI: Confidence interval
ERM: Epiretinal membrane
IOP: Intraocular pressure
IQR: Interquartile range
LNs: Lisch nodules
NF1: Neurofibromatosis type 1
NF2: Neurofibromatosis type 2
NIH: National Institutes of Health
NIR: Near-infrared reflectance
OCT: Optical coherence tomography
RAHs: Retinal astrocytic hamartomas
RR: Rate ratio
RVAs: Retinal vascular abnormalities
SD: Standard deviation
UWF: Ultra-widefield
VA: Visual acuity
