# Supplementary material for: Uveal and retinal abnormalities in an Asian neurofibromatosis type 1 cohort: a cross-sectional study with age-stratified analysis
Source: Eye Vis (Lond). 2026 May 13;13:20. doi: 10.1186/s40662-026-00488-y (PMC13169606; doi:10.1186/s40662-026-00488-y)

**Supplementary Material**

**Table S1.** Pairwise comparisons of Lisch nodules count in iris quadrants

| Pairwise comparisons | Rate ratio (RR) | RR_95%CI | *P** |
| --- | --- | --- | --- |
| IN/IT | 0.83 | (0.71, 0.98) | **0.014** |
| IN/SN | 1.95 | (1.62, 2.36) | **< 0.001** |
| IN/ST | 1.62 | (1.35, 1.94) | **< 0.001** |
| IT/SN | 2.35 | (1.95, 2.82) | **< 0.001** |
| IT/ST | 1.94 | (1.63, 2.32) | **< 0.001** |
| SN/ST | 0.83 | (0.67, 1.02) | 0.090 |

IN = inferior nasal; IT = inferior temporal; SN = superior nasal; ST = superior temporal; CI = confidence interval. * Bonferroni-adjusted. *P* values in bold indicate statistical significance.

**Table S2.** Paired contingency table for Lisch nodules and choroidal abnormalities

|  | Choroidal abnormalities | | |
| --- | --- | --- | --- |
| Lisch nodules |  | 0 | 1 |
|  | 0 | 17  (10.06%) | 16  (9.47%) |
|  | 1 | 0  (0.00%) | 136  (80.47%) |

**Figure S1. Flowchart of participant enrollment and ophthalmic examinations** A total of 231 patients were recruited; two did not meet the diagnostic criteria and one had missing diagnostic information; 228 patients with neurofibromatosis type 1 (NF1) were finally included. Visual acuity (VA) assessment was conducted in 440 eyes of 220 patients, and intraocular pressure (IOP) measurement was completed in 451 eyes of 226 patients. Slit-lamp examination was successfully performed in 454 eyes of 227 patients, with binocular Lisch nodule (LN) counts available in 368 eyes of 184 patients (three eyes excluded due to corneal opacity and two eyes due to poor photographic quality). LN distribution/colour records were available in 350 eyes of 187 patients (24 patients had monocular records and two eyes had poor photographic quality). Ultra-widefield (UWF) fundus imaging was completed in 435 eyes of 218 patients. Near-infrared reflectance (NIR) imaging was conducted in 334 eyes of 169 patients, with binocular choroidal abnormality (CA) counts/areas available in 246 eyes of 123 patients with high-quality NIR images of the posterior pole and quadrants. Distribution records were available in 254 eyes of 136 patients with acceptable NIR images allowing CA identification in each fundus region. Optical coherence tomography (OCT) was conducted in 282 eyes of 145 patients.


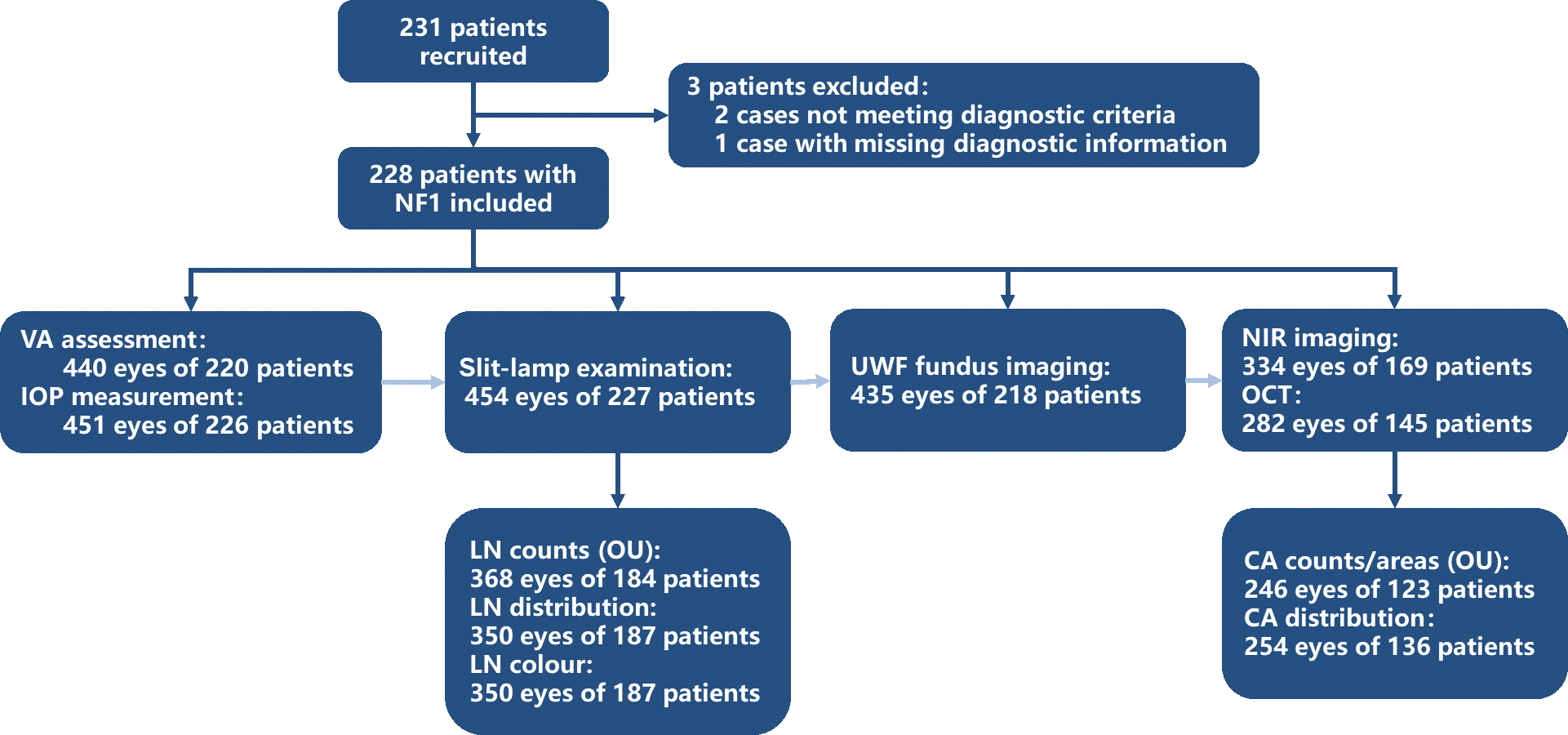


**Figure S2. Number of Lisch nodules by laterality.** Box plots show the median, interquartile range (IQR), and data range of Lisch nodule counts in the right and left eyes. Overlaid jittered points denote individual eye-level raw data, with grey lines linking paired eyes from the same patient. No significant difference in total number of Lisch nodules was observed between the right and left eyes. Paired Wilcoxon rank–sum test, *P* = 0.635. OD, oculus dexter (right eye); OS, oculus sinister (left eye).


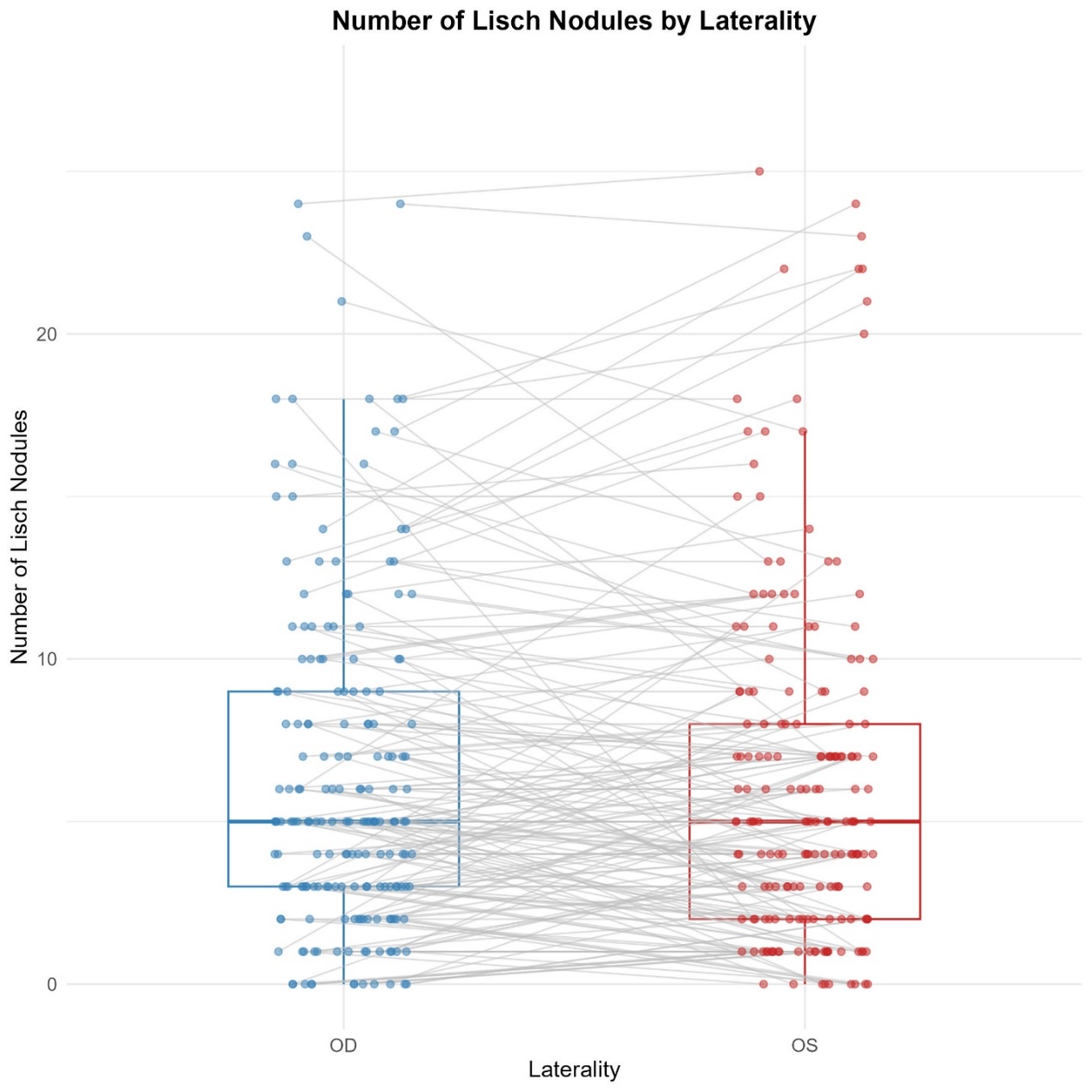


**Figure S3. Number of choroidal abnormalities by laterality.** Box plots show the median, interquartile range (IQR), and data range of choroidal abnormality counts in right and left eyes. Overlaid jittered points denote individual eye-level raw data, with grey lines linking paired eyes from the same patient. No significant difference in total number of choroidal abnormalities was observed between the right and left eyes (paired Wilcoxon rank-sum test, *P* = 0.721). OD, oculus dexter (right eye); OS, oculus sinister (left eye).


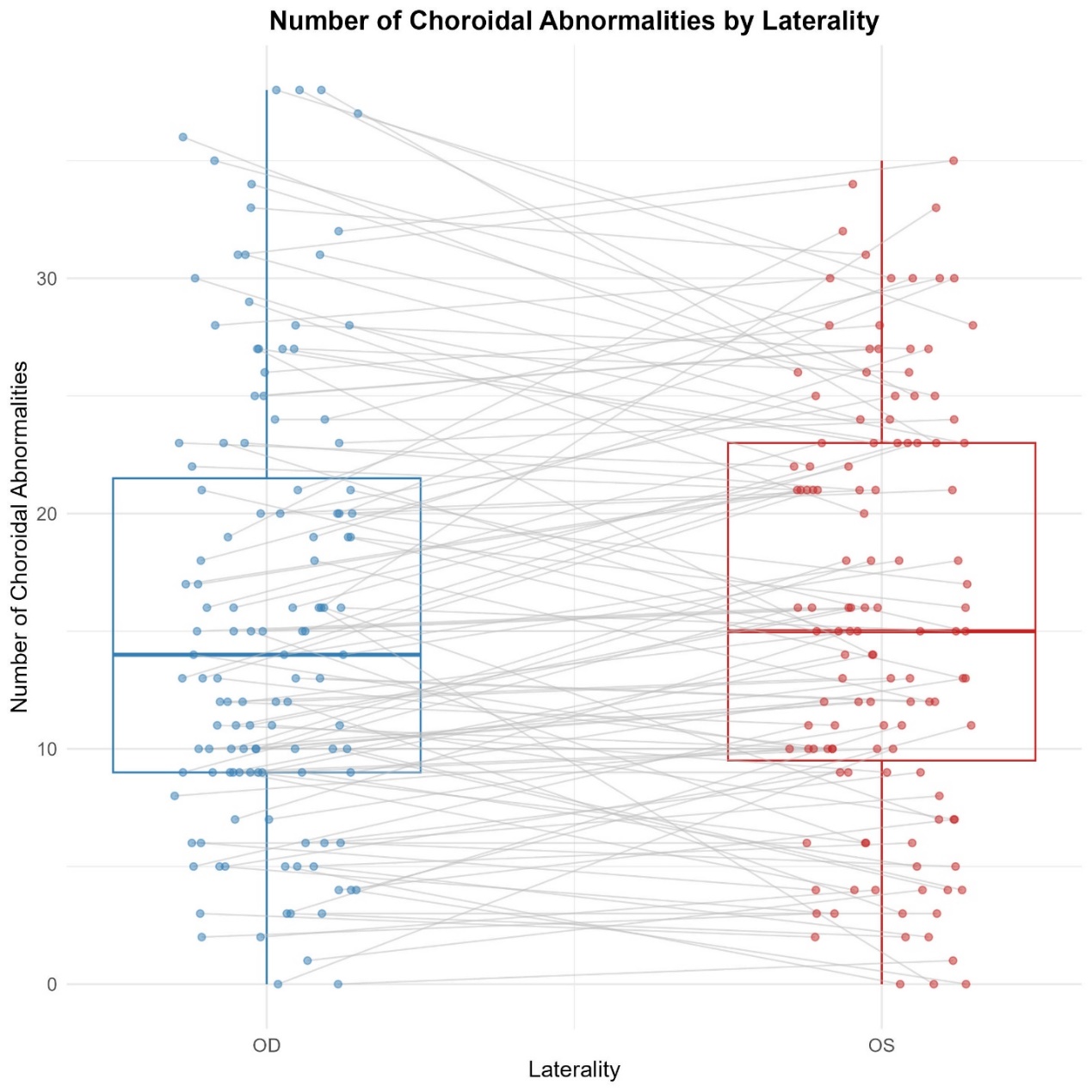


**Figure S4. Area of choroidal abnormalities by laterality.** Box plots show the median, interquartile range (IQR), and data range of choroidal abnormality area in right and left eyes. Overlaid jittered points denote individual eye-level raw data, with grey lines linking paired eyes from the same patient. No significant difference was observed between right and left eyes (paired Wilcoxon rank-sum test, *P* = 0.324). OD, oculus dexter (right eye); OS, oculus sinister (left eye).


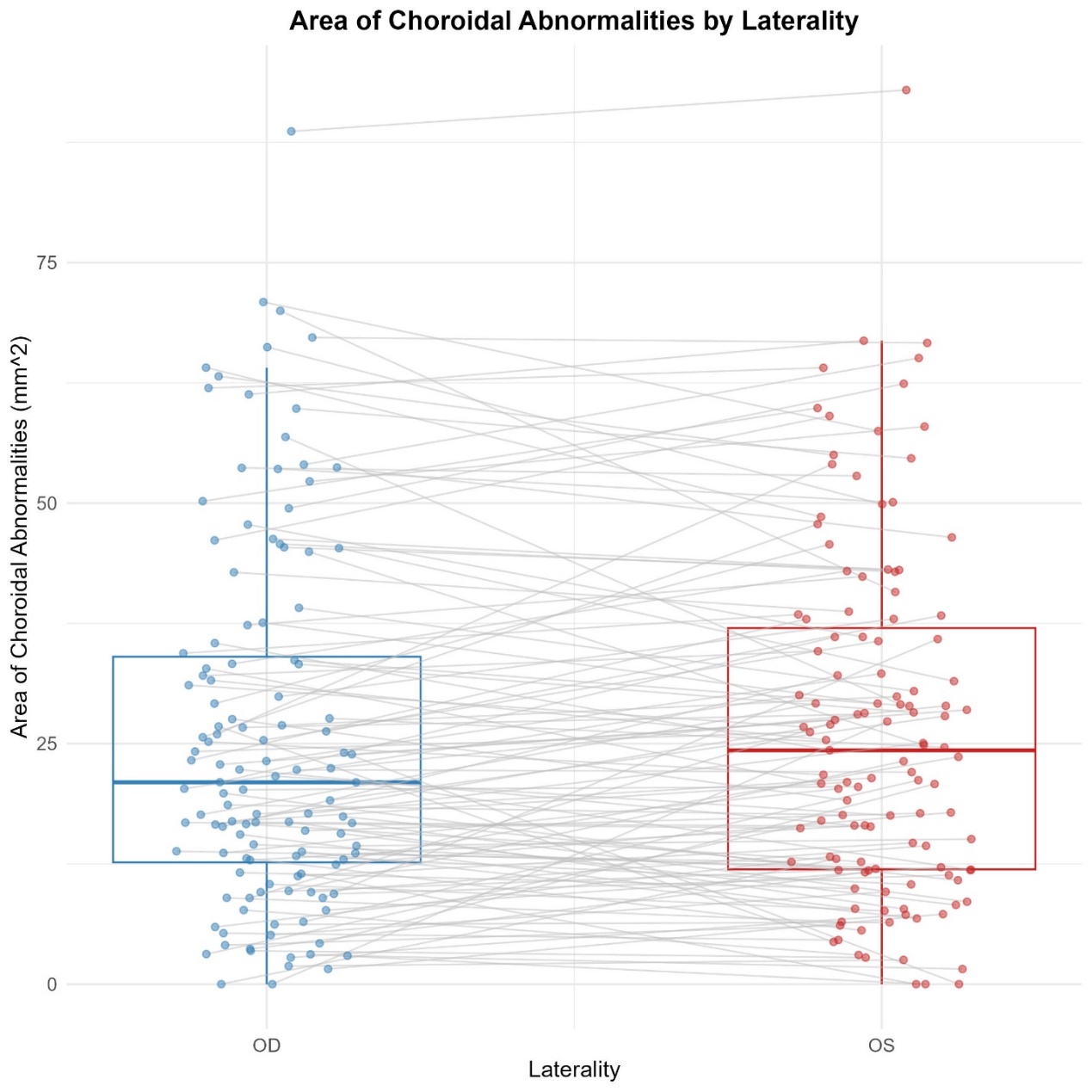


**Figure S5. Prevalence of choroidal abnormalities in each fundus zone.** Zone I showed the highest prevalence (99.2%), followed by zone VI and zone II (81.1% and 79.9%, respectively). Zone III and zone V showed relatively lower prevalence (74.0% and 69.7%, respectively). Chi-square test with Benjamini–Hochberg correction used for multiple comparisons. **P* < 0.05, ***P* < 0.01, ****P* < 0.001.


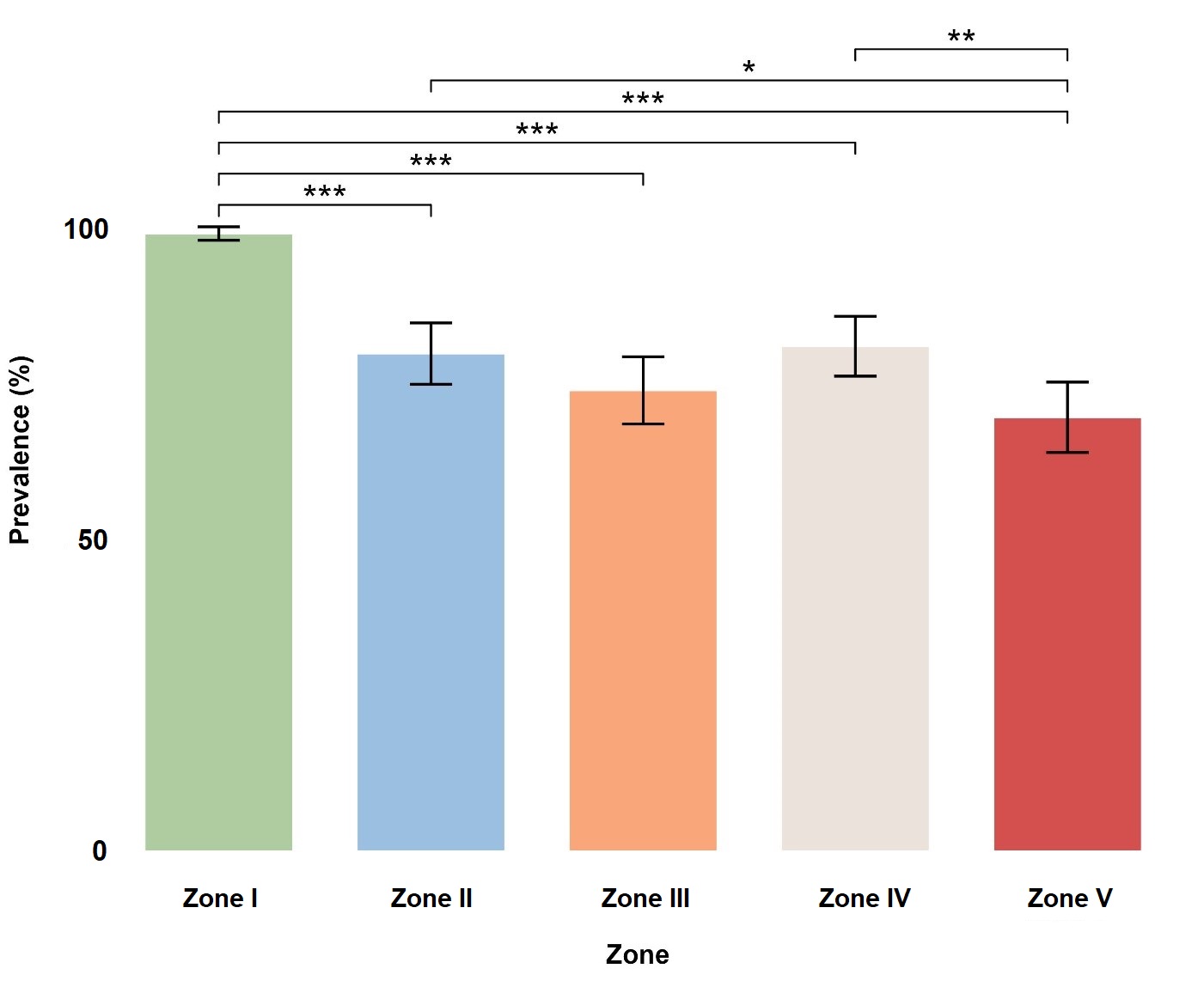


**Figure S6. Prevalence of Lisch nodules in different age groups after excluding patients with mosaic neurofibromatosis type 1 (NF1).** One, three, four, and four patients with mosaic NF1 were excluded from the 0–6 year, 7–12 year, 19–30 year, and 31–70 year groups, respectively. The resulting prevalence were 69.0%, 82.8%, 100.0%, 91.3%, and 89.7%. Fisher’s exact test with Benjamini–Hochberg correction used for multiple comparisons. **P* < 0.05.


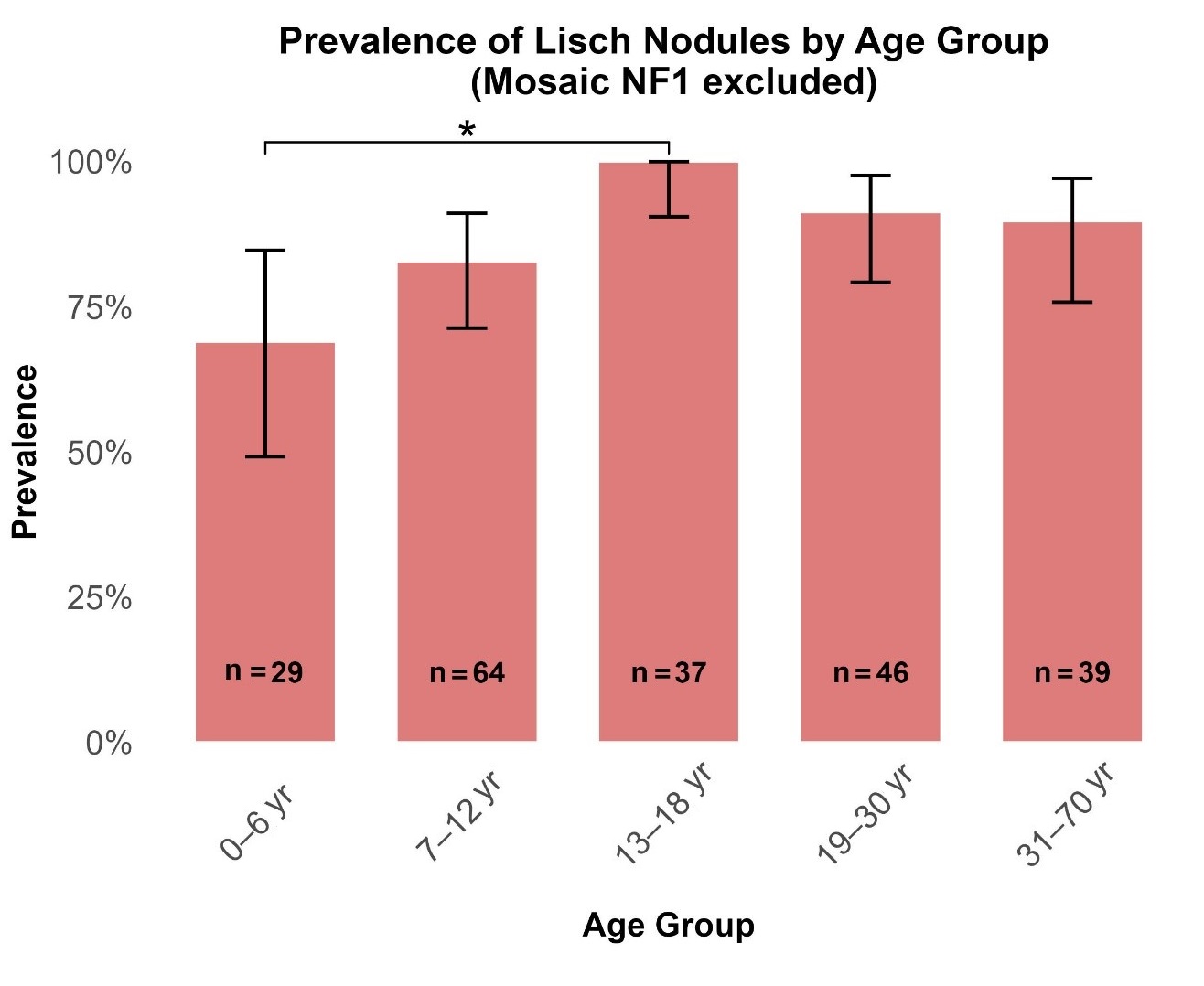

Supplement: Supplementary file 1 — Supplementary Material 1. [file 40662_2026_488_MOESM1_ESM.docx]
